# PUG-View: programmatic access to chemical annotations integrated in PubChem

**DOI:** 10.1186/s13321-019-0375-2

**Published:** 2019-08-09

**Authors:** Sunghwan Kim, Paul A. Thiessen, Tiejun Cheng, Jian Zhang, Asta Gindulyte, Evan E. Bolton

**Affiliations:** National Center for Biotechnology Information, National Library of Medicine, National Institutes of Health, Department of Health and Human Services, 8600 Rockville Pike, Bethesda, MD 20894 USA

**Keywords:** PubChem, PUG-View, PUG-REST, Programmatic access, Web service

## Abstract

PubChem is a chemical data repository that provides comprehensive information on various chemical entities. It contains a wealth of chemical information from hundreds of data sources. Programmatic access to this large amount of data provides researchers with new opportunities for data-intensive research. PubChem provides several programmatic access routes. One of these is PUG-View, which is a Representational State Transfer (REST)-style web service interface specialized for accessing annotation data contained in PubChem. The present paper describes various aspects of PUG-View, including the scope of data accessible through PUG-View, the syntax for formulating a PUG-View request URL, the difference of PUG-View from other web service interfaces in PubChem, and its limitations and usage policies.

## Introduction

PubChem (https://pubchem.ncbi.nlm.nih.gov) [1–3] is a chemical data repository and open chemistry database that aims to provide comprehensive information on various chemical entities, including small molecules, siRNAs, miRNAs, carbohydrates, lipids, peptides, and chemically modified macromolecules. It is one of the most popular chemical information resources in the public domain, with millions of unique users per month. An overview of PubChem, including data sources, data contents, data organization, web-based tools and services, and programmatic access, is given in our previous papers [1, 2].

PubChem contains a wealth of knowledge from hundreds of data sources, which are listed in the PubChem Sources page (https://pubchem.ncbi.nlm.nih.gov/sources). Programmatic access to this large amount of data provides researchers with new opportunities for data-intensive research. PubChem provides several programmatic access routes [4], including NCBI’s Entrez Utilities (E-Utilities) [5], Power User Gateway (PUG) [4], PUG-SOAP [4], and PUG-REST [4, 6, 7]. Among these, PUG-REST [4, 6, 7] is the most heavily used, with millions of daily requests from tens of thousands of unique IP addresses. PUG-REST is a Representational State Transfer (REST) [8, 9]—like web service interface to PubChem. It is designed to handle synchronous tasks, in which the output of the requests is returned to the user immediately, as opposed to a queuing system that requires the use of a polling scheme to check for completion. In addition, because (almost) all necessary information for a PUG-REST request is encoded into a single-line uniform resource locator (URL), it is easy to use and learn relative to other programmatic access interfaces to PubChem that require prior knowledge of a PubChem-specific eXtensible Markup Language (XML) specification [10] or use of a Simple Object Access Protocol (SOAP) envelope [11].

While a substantial amount of PubChem data is from voluntary data submission by individual data depositors, PubChem also collects information from authoritative and manually curated third-party information sources—what PubChem calls annotations. These primarily textual, non-archival annotations cover a wide range of subject fields, including pharmacology, drug target information, toxicology, safety and handling information, patents, environmental health, regulatory requirements, and many others. They are presented in PubChem web pages, such as the Compound Summary page for a given chemical, which provides a comprehensive and aggregated view of the information available in PubChem for that chemical [2]. Because the existing web service interfaces in PubChem (including PUG-REST) were not designed to handle this type of data appropriately, PubChem developed a new REST-style interface, called PUG-View, which serves information needed to render interactive web pages but that also allows one to programmatically access the chemical annotations and summary information in PubChem. The present paper will describe important aspects of PUG-View, including the scope of data accessible through PUG-View, the syntax for formulation a PUG-View request URL, difference of PUG-View from other web service interfaces in PubChem, and its limitations and usage policies.

## Construction and content

### PUG-View URL syntax

**Fig. 1:**
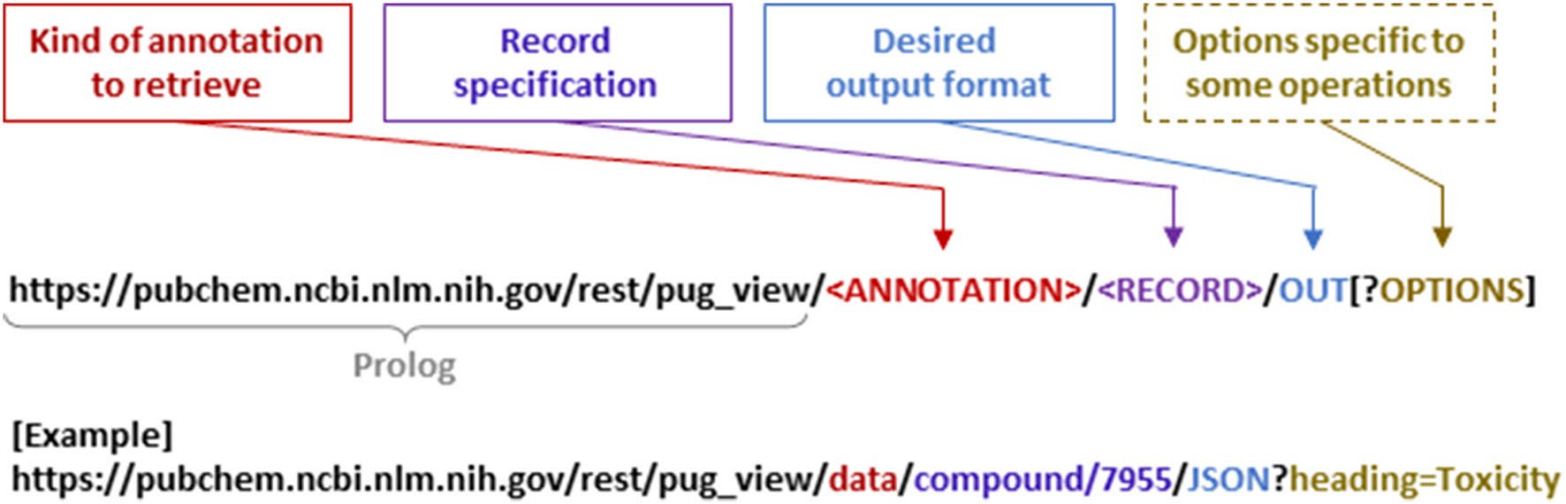
URL construction of a typical PUG-View request. A typical PUG-View request URL encodes three pieces of information (the kind of annotation to retrieve, the record specification, and the desired output format) into one-line URL. Optional parameters specific to some requests may be added to the URL after the question mark (“?”). The available values for the **⟨**ANNOTATION**⟩** part are “data”, “index”, “annotations”, “categories”, “neighbors”, “literature”, “structure”, “image”, “qr”, and “linkout”

Figure 1 shows the general URL syntax for a PUG-View request. With a few exceptions (to be discussed later), almost all PUG-View requests require three pieces of information: (1) the type of annotations to retrieve, (2) the specification of the record of interest, and (3) the desired output format. In PUG-View, these three pieces are encoded in a single, one-line URL. Some requests need additional information, which can be specified as optional parameters (Table 1).

**Table 1:** Optional parameters used in PUG-View requests

Parameter	Value	Description
Page	*n* (integer)	Retrieves the *n*th page of the output data when the data is paginated. This option is useful when downloading the annotations under a specific heading for all compounds
Version	*n* (for a substance) *n.m* (for an assay)	Retrieves the data for a particular version of a substance or assay record. A version number for a substance is an integer. A version number for an assay record is in the form of *n*.*m* (where both *n* and *m* are integers)
Heading	(Sub)heading name	Retrieves data presented in a specified (sub)heading. If the (sub)heading name provided as an optional parameter (i.e., as a URL argument) contains the “space” character, it may be replaced with a plus (“+”) character
Source	Data source name	Retrieves annotation data from a specified source
Callback	func	JSONP callback function name, used mainly in dynamic data retrieval by JavaScript
Response_type	Save or display	Save the returned data (for response_type = save) or displays them on the web browser (for response_type = display)
Toc	TOC name	Retrieves annotation data organized differently than the default for a given record; such as the PubChem Laboratory Chemical Safety Summary (for TOC name = LCSS)

PUG-View provides annotation data in several formats, including XML [10], JavaScript Object Notation (JSON) [12], JSON with padding (JSONP) [13], or Abstract Syntax Notation One (ASN.1) [14, 15] as text (ASNT) or binary (ASNB). Non-textual annotations (e.g., images) may be present in other formats, such as Scalable Vector Graphics (SVG) [16] or Portable Network Graphics (PNG) [17]. The XML schema for the PUG-View annotation data is available at the following URL:

https://pubchem.ncbi.nlm.nih.gov/pug_view/pug_view.xsd.

Note that the JSON and ASN.1 formats follow the same content model, but we do not provide a JSON schema or hyper-schema.

More detailed explanation on how to formulate PUG-View request URLs for specific tasks is provided below, along with examples.

### Full (text) annotations for a given record

As explained elsewhere [2], PubChem data are organized into three inter-linked databases: Compound, Substance, and BioAssay, and each record in these databases is assigned a numeric identifier [called Compound ID (CID), Substance ID (SID), or Assay ID (AID), depending on the type of the record]. Each record has a dedicated web page that presents information available for that record in PubChem. This page is called the Compound Summary, Substance Record, or BioAssay Record page, depending on the type of the record [2]. The annotations presented on this page can be retrieved using the following URLs (with CID 1983, SID 46506142, and AID 1259416 as examples).

- For the Compound Summary page (CID 1983)
https://pubchem.ncbi.nlm.nih.gov/rest/pug_view/data/compound/1983/JSON
- For the Substance Record page (SID 46506142)
https://pubchem.ncbi.nlm.nih.gov/rest/pug_view/data/substance/46506142/JSON
- For the BioAssay Record page (AID 1259416)
https://pubchem.ncbi.nlm.nih.gov/rest/pug_view/data/assay/1259416/JSON

It is noteworthy that only a single record can be encoded in a PUG-View request URL. PUG-View is also used as the backend service to retrieve the data that are presented on the Summary page for a given compound (or the Record page for a given substance or bioassay), and so is optimized for individual record retrieval, rather than as a bulk service.

The PubChem Laboratory Chemical Safety Summary (LCSS) [18, 19] provides a concise view of the health and safety data for a given compound. The data presented on the LCSS page is a subset of those presented on the Compound Summary page of the corresponding chemical. One can retrieve these LCSS data by using an optional parameter to PUG-View (Table 1), as shown in this example:

https://pubchem.ncbi.nlm.nih.gov/rest/pug_view/data/compound/887/JSON?toc=LCSS+TOC.

This example returns the health and safety data for CID 887 (methanol).

It is possible to retrieve a previous version of depositor-provided data for a substance record or assay record by specifying the version number as an optional parameter (Table 1), as shown in the following examples:

https://pubchem.ncbi.nlm.nih.gov/rest/pug_view/data/substance/46507068/JSON?version=2,

https://pubchem.ncbi.nlm.nih.gov/rest/pug_view/data/assay/369/JSON?version=1.1

In addition to the Summary and Record pages, PubChem provides data-centric view pages, including the Patent View and Target View pages [1]. The Patent View page for a given patent provides compounds and substances mentioned in it, along with other information including patent title, abstract, application/publication dates, applicant and inventor. The Target View page for a given gene or protein provides a “target-centric” view of PubChem data pertinent to a given gene or protein target, including the chemicals tested against the target and biological assay experiments performed against the target, along with the annotated information about the target collected from authoritative sources. The annotated information presented on these View pages are also available for programmatic access through PUG-View, as shown in the examples below:

- Patent View (US4681893)
https://pubchem.ncbi.nlm.nih.gov/rest/pug_view/data/patent/US4681893/JSON
- Gene Target View (Gene ID 3269)
https://pubchem.ncbi.nlm.nih.gov/rest/pug_view/data/gene/3269/JSON
- Protein Target View (accession P35367)
https://pubchem.ncbi.nlm.nih.gov/rest/pug_view/data/protein/P35367/JSON

Note that the request URLs for the gene and protein targets use the NCBI Gene ID (3269) and protein accession identifier (P35367), respectively, which correspond to the human histamine receptor H1 (HRH1) gene and its encoded protein.

### Particular type of (text) annotations for a given record

The annotations presented under a given (sub)heading of the Summary/Record or View page can be retrieved by specifying the (sub)heading of interest as an optional parameter (Table 1). For example, the following two URLs retrieve the annotations specified:

- Experimental properties of CID 1983
https://pubchem.ncbi.nlm.nih.gov/rest/pug_view/data/compound/1983/JSON?heading=Experimental+Properties
- Solubility of CID 1983
  https://pubchem.ncbi.nlm.nih.gov/rest/pug_view/data/compound/1983/JSON?heading=Solubility.

Note that the space between the two words in “Experimental Properties” is replaced with the “+” character (this is standard URL encoding). Section headings that can be used in PUG-View data retrieval can be found in the PubChem Compound TOC tree (using the PubChem Classification Browser [2]: 

https://pubchem.ncbi.nlm.nih.gov/classification/#hid=72).

The above two PUG-View request URLs assume that CID 1983 has annotations in the “Experimental Properties” and “Solubility” sections. If the compound does not have any data to present in that section, the requests would return an error message. The presence (or absence) of the desired annotations for a given compound can be checked through a PUG-View request that retrieves the available (sub)headings for that compound without getting all annotations. This can be done using the following URL (with CID 1983 as an example):

- Available (sub)headings (“indices”) for CID 1983
https://pubchem.ncbi.nlm.nih.gov/rest/pug_view/index/compound/1983/JSON

Essentially, this request returns the Table of Contents for the Summary page of CID 1983, without the entire data content of the record.

### Particular type of (text) annotation for all records

PUG-View allows users to retrieve a specific type of annotation for all records. For example, the following URLs allow one to download all viscosity measurements, with links to primary PubChem records:

https://pubchem.ncbi.nlm.nih.gov/rest/pug_view/annotations/heading/Viscosity/JSON,

https://pubchem.ncbi.nlm.nih.gov/rest/pug_view/annotations/heading/JSON?heading=Viscosity.

The two URLs are equivalent to each other, returning the same result. The heading name in the URL is case-*insensitive*. However, if the heading name contains a special character that is not compatible with the URL syntax (e.g., a forward slash), the heading must be provided as an optional parameter (Table 1):

https://pubchem.ncbi.nlm.nih.gov/rest/pug_view/annotations/heading/JSON?heading=formulations/preparations.

Note that the request URLs for bulk download of specific annotation data are exceptions to the URL syntax presented in Fig. 1, in the sense that these URLs do not contain a specific input record identifier.

Some annotation data in PubChem are from multiple data sources, and it is possible to retrieve the annotation data from a specific data source by providing the source name as an optional parameter (Table 1) in a PUG-View request. For instance, the following request returns the octanol–water partition coefficient (log P) data from DrugBank [20]:

https://pubchem.ncbi.nlm.nih.gov/rest/pug_view/annotations/heading/JSON/?heading=LogP&source=DrugBank.

Some headings have so much annotation data that it is not possible to retrieve all of them through a single PUG-View request. Like PUG-REST, a PUG-View request has a maximum time limit of 30 s. If a PUG-View request exceeds this, a time-out error is returned. To circumvent time-out errors, the returned data have “TotalPage” and “Pages” values provided. These indicate the total page count of the annotations data and the current page number of the returned data, respectively. By default, only the data on the first page is returned and subsequent pages (up to the “TotalPages” limit) can be accessed by adding a page argument (Table 1). For example, the following URL returns page 212 of the CAS registry number data (among the total of > 1000 pages):

https://pubchem.ncbi.nlm.nih.gov/rest/pug_view/annotations/heading/CAS/JSON?page=212.

One should check the “TotalPages” number of the returned annotation data to see if more data is available. In addition, when retrieving annotation data through multiple PUG-View calls, users should throttle their requests not to overload PubChem servers (to be discussed later in more detail). Some fields are too numerous to be downloaded in bulk through PUG-View calls (e.g., synonyms, InChI strings, associated patents for all compounds). Such information should be downloaded via the PubChem FTP site

(ftp://ftp.ncbi.nlm.nih.gov/pubchem/ or https://ftp.ncbi.nlm.nih.gov/pubchem/).

### Special reports

In addition to serving out general record summary data blobs, PUG-View also handles a variety of special reports for more specialized data, each with its own unique data model that is different from the generic summary record. These are described in the following sections.

### Substances by category

**Fig. 2:**
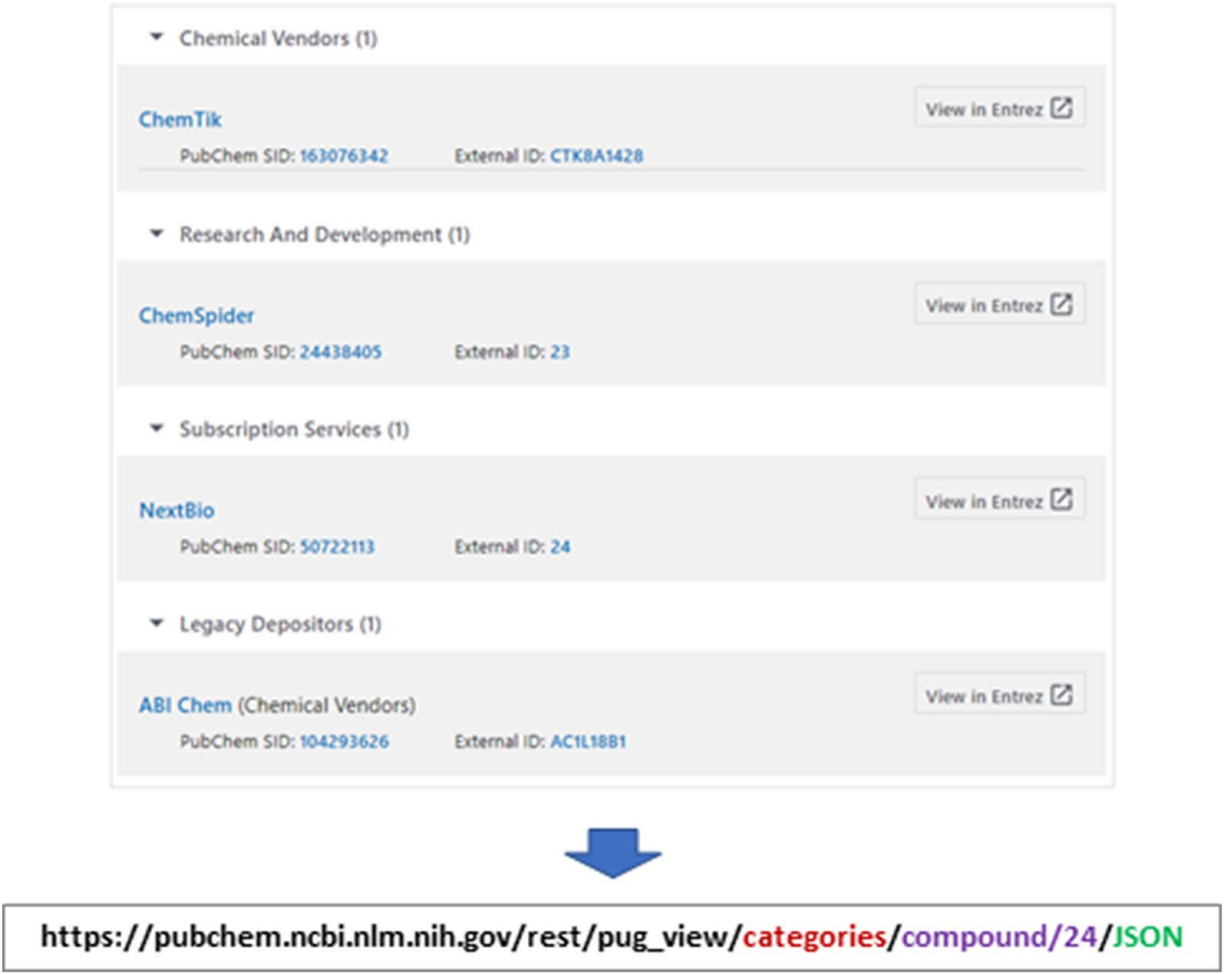
Retrieval of substances associated with a given compound, grouped by source category. The substance records associated with a given compound (CID 24 as an example), presented in the “Substance by Category” section of the Compound Summary page, can be downloaded through PUG-View

The Summary page of each compound has the “Substances by Categories” section, which presents the associated substance records classified according to the source category (Fig. 2). For example, the following URL directs to the “Substances by Categories” section for CID 24. https://pubchem.ncbi.nlm.nih.gov/compound/24#section=Substances-by-Category.

The data presented in this section can be retrieved using the following PUG-View request URL:

https://pubchem.ncbi.nlm.nih.gov/rest/pug_view/categories/compound/24/JSON.

### Related compounds with annotation

For each compound, PubChem pre-computes its “neighbors”, which are structurally similar compounds in terms of 2-D and 3-D similarity measures, as described in detail elsewhere [21–24]. If these neighbors have pre-defined types of annotations (such as medications, literature, 3-D structure, bioactivities, and patents), they are presented in the “Related Compounds with Annotation” section of the Summary page of the compound, as in the following example (for CID 60823):

https://pubchem.ncbi.nlm.nih.gov/compound/60823#section=Related-Compounds-with-Annotation.

The data content presented in this section may be retrieved through PUG-View, using the following request URL:

https://pubchem.ncbi.nlm.nih.gov/rest/pug_view/neighbors/compound/60823/JSON.

Note that this request returns only the neighbors with pre-defined types of annotations. To get all neighbors for a given compound (regardless of whether they have annotations or not), PUG-REST (not PUG-View) should be used as explained in previous papers [4, 6, 7].

### Literature

As explained in a previous paper [25], PubChem contains a large amount of associations between chemicals and scientific articles. Some of these associations are derived by PubChem through matching between chemical names and MeSH terms associated with PubMed articles. The links to such articles for a given compound are available in the “NLM curated PubMed citations” section of the Compound Summary page, as shown in the following example:

https://pubchem.ncbi.nlm.nih.gov/compound/2244#section=NLM-Curated-PubMed-Citations.

Note that the section allows one to retrieve not only all the relevant articles but also those indexed by the National Library of Medicine (NLM) with a particular MeSH subheading. One can get the links shown in this section via the following PUG-View request URL:

https://pubchem.ncbi.nlm.nih.gov/rest/pug_view/literature/compound/2244/JSON.

PubChem also collects some scientific articles for a given compound from curated sources, and they are displayed in several sections of the Compound Summary page, such as the “Synthesis References”, “General References”, and “Metabolite References”. These data can be retrieved through a PUG-View record retrieval with the desired (sub)heading specified as an optional parameter. For example, the following URL retrieves papers about the synthesis of CID 2244 (aspirin), presented in the “Synthesis Reference” section:

https://pubchem.ncbi.nlm.nih.gov/rest/pug_view/data/compound/2244/JSON?heading=Synthesis+References.

Many chemical-literature associations in PubChem are submitted by individual data depositors and presented in the “Depositor Provided PubMed Citations”. These data may be retrieved through PUG-REST (not PUG-View). For example, the following URL retrieves the depositor-provided PubMed Citations for CID 2244:

https://pubchem.ncbi.nlm.nih.gov/rest/pug/compound/cid/2244/xrefs/PubMedID/JSON.

### 3-D protein-bound structures

Some compounds in PubChem have experimental 3-D protein-bound structures, collected from Protein Data Bank (PDB) [26] and Molecular Modeling Database (MMDB) [27]. While MMDB collects and annotates experimental structures deposited in PDB, the two resources provide slightly different sets of protein–ligand associations. PUG-View supports programmatic access to the list of the protein structures associated with a given chemical, collected from MMDB. For example, the following URL retrieves the MMDB protein structures with which CID 123631 has been co-crystalized:

https://pubchem.ncbi.nlm.nih.gov/rest/pug_view/structure/compound/123631/JSON.

### Biologic description

The PubChem Compound database contains more than a half million biologic molecules, including short peptides, carbohydrates, lipids, nucleotides, etc. The Compound Summary page of a biologic displays a two-dimensional (2-D) structure representation in the “Biologic Description” section, as shown in the following example (for CID 91848714):

https://pubchem.ncbi.nlm.nih.gov/compound/91848714#section=Biologic-Description.

**Fig. 3:**
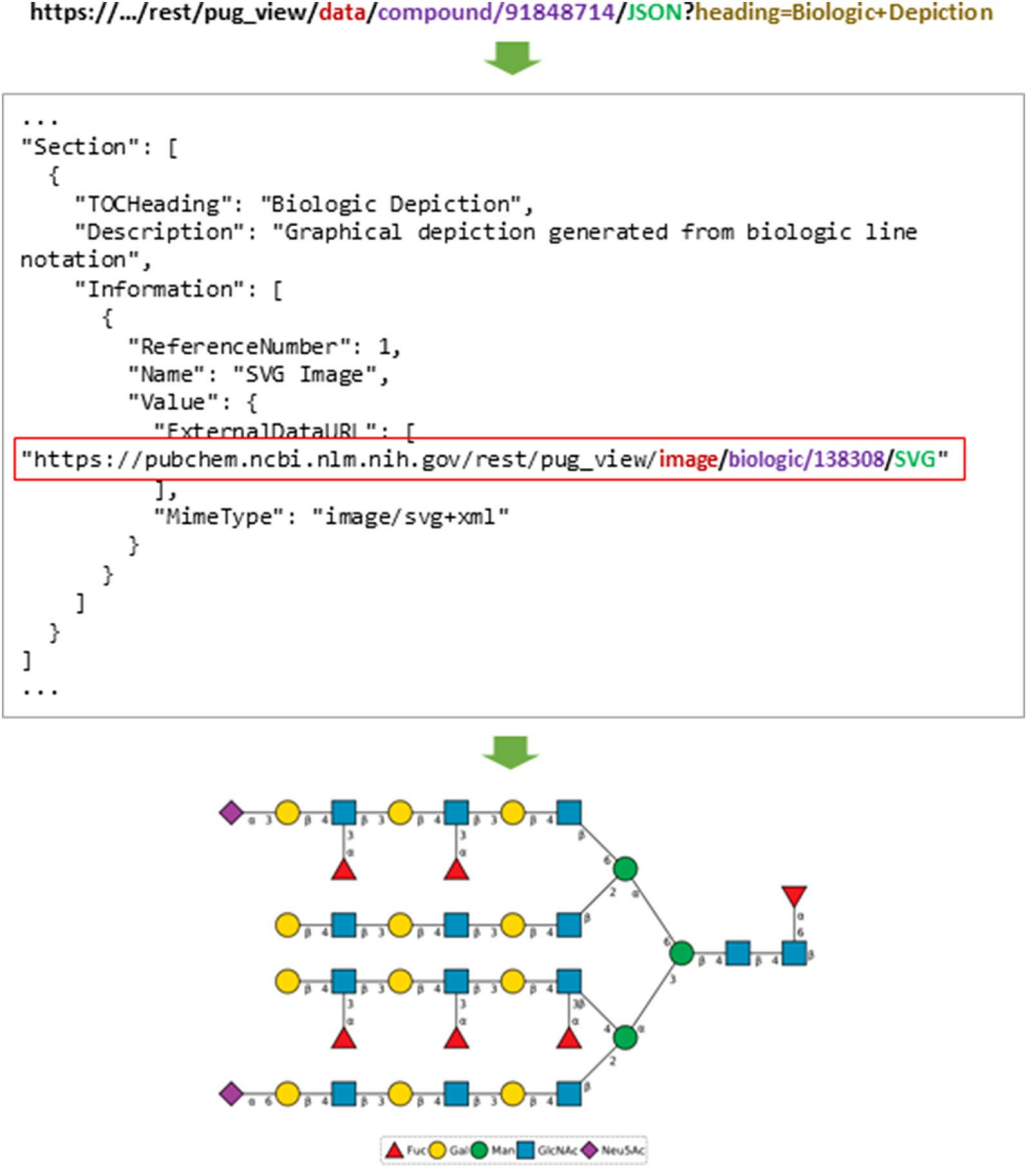
Retrieval of biologic descriptions. To get the biological depiction image of a biologic compound (CID 91848714 as an example), the internal identifier (138308) to the biologic compound is retrieved and then used in a subsequent request for the image. The “…” in the figure above refers to the PubChem web address “http://pubchem.ncbi.nlm.nih.gov”, which is omitted

Retrieval of the biologic depiction image for a compound through PUG-View is a two-step process (Fig. 3). Each biologic molecule is assigned an internal identifier, which can be found in the annotation data in the “Biologic Depiction” section, retrieved from a PUG-View request. For example, the internal biologic identifier for the above biologic structure (CID 91848714) can be retrieved via the URL:

https://pubchem.ncbi.nlm.nih.gov/rest/pug_view/data/compound/91848714/JSON?heading=Biologic+Description.

Then, the retrieved internal identifier (138308) can be used to obtain the biologic depiction image in SVG format, using the following URL:

https://pubchem.ncbi.nlm.nih.gov/rest/pug_view/image/biologic/138308/SVG.

Below is another example of the biologic depiction image retrieval for CID 56842075, whose internal identifier is 703612:

https://pubchem.ncbi.nlm.nih.gov/rest/pug_view/image/biologic/703612/SVG.

Note that the two biologic depiction examples given here return the images of different biologic types: carbohydrate (for CID 91848714) and peptide (for CID 56842075).

### Annotation attachments

Some annotation data in PubChem are files attached to a given record, such as spectral images. For example, CID 7510 has several mass spectral data (including meta data and spectral images). These can be accessed via its Compound Summary page using the URL:

https://pubchem.ncbi.nlm.nih.gov/compound/7510#section=Mass-Spectrometry.

**Fig. 4:**
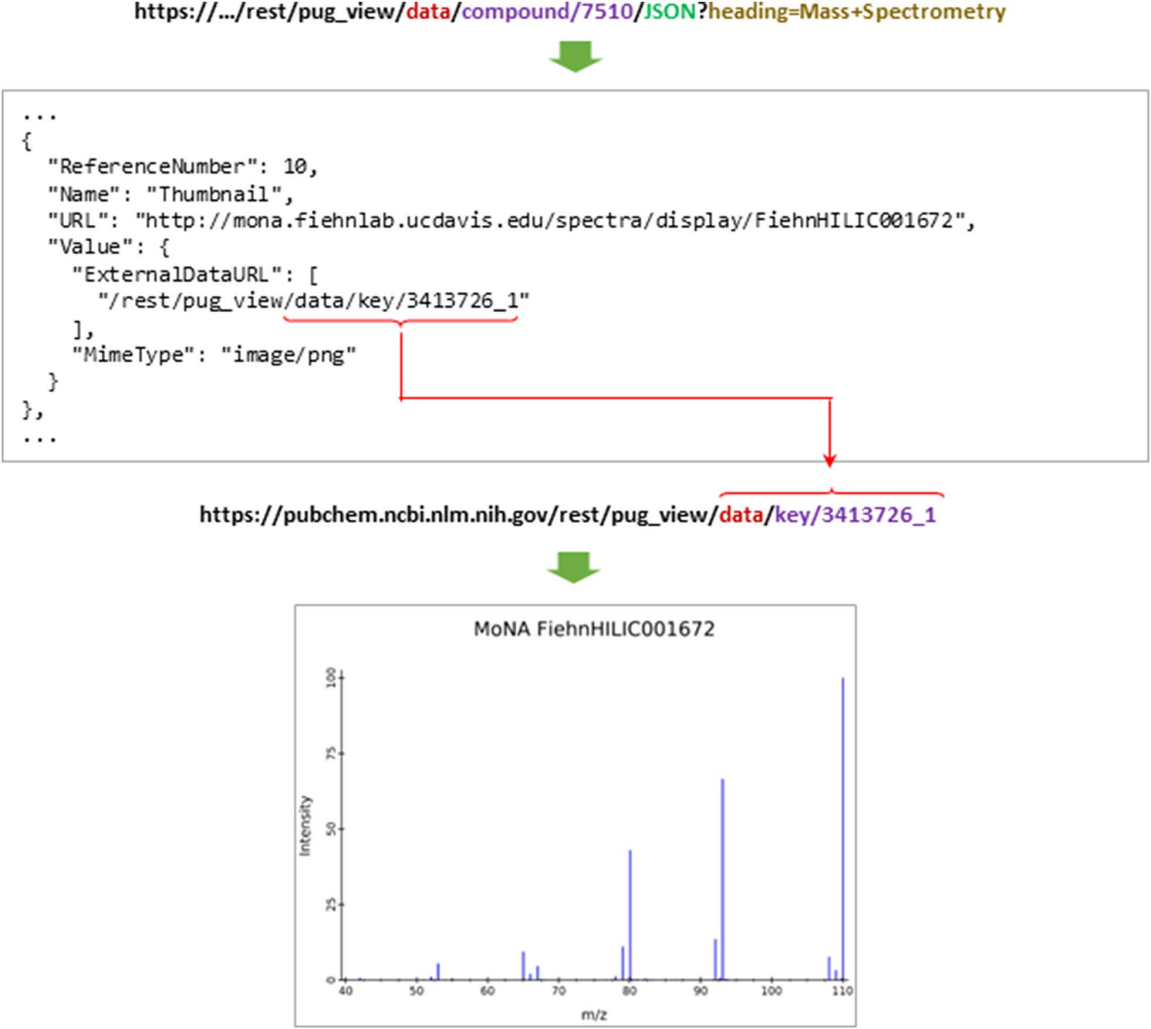
Retrieval of annotation attachments. Some annotations collected from authoritative sources are file attachments. Retrieving these attachments through PUG-View is a two-step process. In a first PUG-View request, the internal identifier for an attachment is obtained. This identifier is used in a second PUG-View request to retrieve the attachment. The “…” in the figure above refers to the PubChem web address “http://pubchem.ncbi.nlm.nih.gov”, which is omitted

Accessing these spectral images through PUG-View is a two-step process (Fig. 4). Each spectral image on this page has a unique key, which can be found in the annotation data returned from the following PUG-View request:

https://pubchem.ncbi.nlm.nih.gov/rest/pug_view/data/compound/7510/JSON?heading=Mass+Spectrometry.

One of the keys from this request is “3413726_1” and it can be used in a subsequent PUG-REST request to retrieve the corresponding image, using the following URL:

https://pubchem.ncbi.nlm.nih.gov/rest/pug_view/data/key/3413726_1.

Note that, because the returned data from this request is an existing file (in the Portable Network Graphics (PNG) format [17]), the output format should not be specified in this PUG-View request URL. (Data annotations like this each have a mime type returned in the Content-Type header of the PUG-View request, that identifies what sort of data is being provided, such as “image/png” for PNG images).

### QR codes

Through PUG-View, one can generate the QR codes that provides a quick access to information for a given compound. For example, the QR codes generated from the following request URLs lead users to the PubChem Compound Summary page for CID 702 (ethanol), either directly or indirectly (via google):

https://pubchem.ncbi.nlm.nih.gov/rest/pug_view/qr/long/compound/702/SVG, https://pubchem.ncbi.nlm.nih.gov/rest/pug_view/qr/short/compound/702/SVG.

The chemical QR code was developed to provide easy and quick access from mobile devices to chemical hazard and safety information for chemicals commonly used in the academic laboratory. It is possible to generate a QR code that encodes the link specifically to the PubChem LCSS page [18, 19] of a chemical, as shown in this example:

https://pubchem.ncbi.nlm.nih.gov/rest/pug_view/qr/long/compound/702/LCSS/SVG.

### Linkout

The NCBI LinkOut service [28] provides links between records in the NCBI resources and those in various information resources beyond the NCBI systems. PUG-View can be used to retrieve a list of all the NCBI LinkOut data available for a compound record, as shown in this example (for CID 2244):

https://pubchem.ncbi.nlm.nih.gov/rest/pug_view/linkout/compound/2244/JSON.

## Utility and discussion

### Limitation of PUG-View

**Fig. 5:**
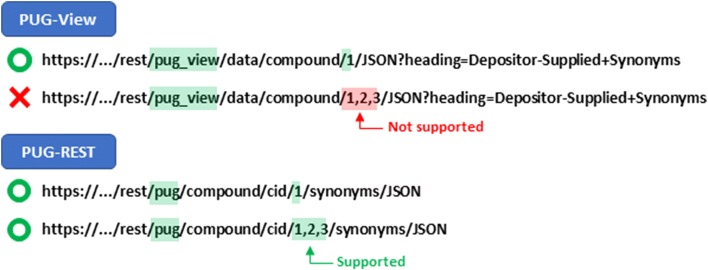
Multiple record identifiers in PUG-View and PUG-REST. While only a single record identifier is supported in the PUG-View request URL, PUG-REST can accept multiple record identifiers. The “…” in the figure above refers to the PubChem web address “http://pubchem.ncbi.nlm.nih.gov”, which is omitted

As mentioned previously, PUG-View is used as a backend service to serve the data presented on the interactive web Summary page of a PubChem record. Because this Summary page presents information on a single record, PUG-View is designed to handle only one input identifier at a time. Therefore, PUG-View cannot take multiple record identifiers in the request URL (see Fig. 5).

In addition, when accessing the annotation data for a compound, PUG-View primarily takes only the corresponding CID (rather than chemical names, International Chemical Identifier (InChI) [29, 30], InChIKeys [29, 30], Simplified Molecular Input Line Entry System (SMILES) [31–33] or other identifiers) in the request URL, because the CID is used as the primary record identifier (accession) for the Compound database. Therefore, to get annotations corresponding to non-CID identifiers, they need to be converted to CIDs first (e.g., using PUG REST) and then those CIDs should be used in PUG-View requests.

It is also noteworthy that not all data presented on the Summary/Record/View page are necessarily available through PUG-View. Some data are available only through PUG-REST (e.g., computed compound properties) or some other backend service. The JSON/XML file returned from a PUG-View request for such data usually have nodes called “ExternalTableName” and “ExternalTableNumRows”, as exemplified in the output returned from the following request:

https://pubchem.ncbi.nlm.nih.gov/rest/pug_view/data/compound/60823/JSON?heading=Depositor+Provided+PubMed+Citations.

Such tables are also available programmatically but are beyond the scope of this paper. The nature of the content (non-archival data from contributors) is what sets PUG View apart from other PubChem content interfaces like PUG REST.

### Comparison of PUG-View with PUG-REST

PUG-View can be easily confused with PUG-REST. Although both PUG-View and PUG-REST are REST-like interfaces, they aim to serve distinct kinds of data in general. PUG-REST primarily provides access to data that can be readily structured, such as computed properties of compounds, activity data for assays, associations (cross-references) between PubChem records and other resources, and so on. On the other hand, PUG-View is intended to support the retrieval of unstructured, largely textual annotation data (e.g., excerpts about handling or first aid procedure for a chemical). The data models for the JSON/XML returned by these services are also completely different. In general, PUG-REST is intended to be used to grab small, specific bits of information; whereas PUG-View is used for larger reports, another reason that PUG-View does not handle multiple records in a single request.

### Usage policies

All PUG-View requests are subject to a 30-s time limit. If a request takes longer than this 30-s limit for any reason, it will return an error message. Because this time limit would be an issue when downloading a large annotation data (e.g., specific annotation for all records), it is “paginated” and should be retrieved through multiple requests using a loop.

PubChem often receives too many programmatic service requests (including PUG-REST and PUG-View), causing service interruptions. Therefore, as described elsewhere [6], PubChem has usage policies on request volume limits. The current limits are:

- No more than 5 requests per second.
- No more than 400 requests per minute.
- No longer than 300 s running time per minute.

Note that, when PubChem gets an excessive number of service requests, these limits are tightened through a dynamic web-request throttling [6]. Users should moderate the speed at which requests are sent according to the traffic status information contained in the HTTP response header returned for PUG-View and PUG-REST requests. More detailed information can be found at the PubChem Help page

(https://pubchemdocs.ncbi.nlm.nih.gov/programmatic-access$_RequestVolumeLimtations).

## Conclusions

Third-party annotation content in PubChem has an ever-expanding scope and is increasingly provided for entities beyond the archival records [assay (AID), substance (SID), and compound (CID)], including the recently released protein target, gene target, and patent data view pages. PUG-View allows one to programmatically access this annotation content in PubChem and is also used as a backend service to provide annotation content to display on the web Summary pages for PubChem records. PUG-View is available to the public free of charge.

Although both PUG-View and PUG-REST are REST-style interfaces, they serve different types of data and have different use cases. For example, while PUG-REST is best for accessing specific individual computed compound properties, PUG-View is geared towards retrieving full textual annotations and other longer specialized reports.

PUG-View has several limitations. For example, a PUG-View request cannot take multiple input record identifiers. In addition, annotation data under multiple headings for a given compound cannot be retrieved with a single request, unless the full annotation data retrieval is requested. Importantly, some specialized (especially tabular) data come from other backend service(s) and are not currently accessible through PUG-View.

## Data Availability

PUG-View is provided to the public free of charge.

## Abbreviations

PUG: Power User Gateway
REST: Representational State Transfer [8, 9]
SOAP: Simple Object Access Protocol [11]
XML: eXtensible Markup Language [10]
URL: uniform resource locator
JSON: JavaScript Object Notation [12]
JSONP: JavaScript Object Notation with padding [13]
SVG: Scalable Vector Graphics [16]
ASN.1: Abstract Syntax Notation One [14]
CID: Compound ID
SID: Substance ID
AID: Assay ID
PNG: Portable Network Graphics [17]
LCSS: Laboratory Chemical Safety Summary [18, 19]
SMILES: Simplified Molecular Input Line Entry System [31–33]
InChI: International Chemical Identifier [29, 30]
